# Medical masks vs N95 respirators for preventing COVID‐19 in healthcare workers: A systematic review and meta‐analysis of randomized trials

**DOI:** 10.1111/irv.12745

**Published:** 2020-04-21

**Authors:** Jessica J. Bartoszko, Mohammed Abdul Malik Farooqi, Waleed Alhazzani, Mark Loeb

**Affiliations:** ^1^ Department of Health Research Methods, Evidence and Impact McMaster University Hamilton ON Canada; ^2^ Department of Medicine Division of Respirology McMaster University Hamilton ON Canada; ^3^ Department of Medicine Division of Critical Care McMaster University Hamilton ON Canada; ^4^ Department of Pathology and Molecular Medicine McMaster University Hamilton ON Canada

**Keywords:** coronavirus, COVID‐19, masks, meta‐analysis, N95 respirators, SARS‐CoV‐2, systematic review

## Abstract

**Background:**

Respiratory protective devices are critical in protecting against infection in healthcare workers at high risk of novel 2019 coronavirus disease (COVID‐19); however, recommendations are conflicting and epidemiological data on their relative effectiveness against COVID‐19 are limited.

**Purpose:**

To compare medical masks to N95 respirators in preventing laboratory‐confirmed viral infection and respiratory illness including coronavirus specifically in healthcare workers.

**Data Sources:**

MEDLINE, Embase, and CENTRAL from January 1, 2014, to March 9, 2020. Update of published search conducted from January 1, 1990, to December 9, 2014.

**Study Selection:**

Randomized controlled trials (RCTs) comparing the protective effect of medical masks to N95 respirators in healthcare workers.

**Data Extraction:**

Reviewer pair independently screened, extracted data, and assessed risk of bias and the certainty of the evidence.

**Data Synthesis:**

Four RCTs were meta‐analyzed adjusting for clustering. Compared with N95 respirators; the use of medical masks did not increase laboratory‐confirmed viral (including coronaviruses) respiratory infection (OR 1.06; 95% CI 0.90‐1.25; *I* ^2^ = 0%; low certainty in the evidence) or clinical respiratory illness (OR 1.49; 95% CI: 0.98‐2.28; *I* ^2^ = 78%; very low certainty in the evidence). Only one trial evaluated coronaviruses separately and found no difference between the two groups (*P* = .49).

**Limitations:**

Indirectness and imprecision of available evidence.

**Conclusions:**

Low certainty evidence suggests that medical masks and N95 respirators offer similar protection against viral respiratory infection including coronavirus in healthcare workers during non–aerosol‐generating care. Preservation of N95 respirators for high‐risk, aerosol‐generating procedures in this pandemic should be considered when in short supply.

## 1 INTRODUCTION

Novel 2019 coronavirus disease (COVID‐19) was declared a pandemic by the World Health Organization (WHO) on March 11, 2020, after the identification of >118 000 cases in 114 countries.^1^ As government officials and public health stakeholders implement measures to slow the spread of SARS‐CoV‐2, healthcare workers treating COVID‐19 patients are among those at highest risk of infection. During the severe acute respiratory disease (SARS) pandemic in 2003, healthcare workers made up 21% (1706/8096) of global cases.^2^ An early report from a single‐center case series of 138 hospitalized patients for COVID‐19 in Wuhan, China in January 2020 found that 29% (40/138) were healthcare workers that had been infected in hospital.^3^ As of February 11, 2020, China's Infectious Disease Information System has reported COVID‐19 in 1716 healthcare workers.^4^

Although the transmission of COVID‐19 is not yet fully understood, it is believed to be mainly through large respiratory droplets.^5^ For aerosol‐generating procedures, such as intubation or bronchoscopy, there is consensus that N95 respirators offer better protection than medical masks.^6^, ^7^, ^8^, ^9^ N95 respirators are designed to minimize facial seal leakage because of tight fit and prevent inhalation of small airborne particles. They also are required to pass filtration tests. In contrast, medical masks (also known as surgical masks) are loose fitting, provide barrier protection against large droplets and prevent hand‐to‐face contact.^10^

Globally, current recommendations to protect healthcare workers against COVID‐19 for non–aerosol‐generating care are conflicting.^6^, ^7^, ^8^, ^9^ For example, the U.S Centers for Disease Control and Prevention (CDC) and European Centre for Disease and Prevention (ECDC) recommend the N95 respirator for non–aerosol‐generating routine care of patients with COVID‐19,^6^, ^7^ while the World Health Organization and the Public Health Agency of Canada recommend medical masks.^8^, ^9^

Shortages of personal protective equipment for healthcare workers, including medical masks and N95 respirators, have been widely reported in this pandemic.^11^ A shortage of N95 respirators for aerosol‐generating procedures, where the risk to healthcare workers is high, is of particular concern. This, along with conflicting recommendations, warrants an update of previous systematic reviews (where the last search was conducted in 2015).^12^, ^13^ Evidence to support similar relative effectiveness of medical masks compared with N95 respirators might help preserve stockpiles of N95 respirators for aerosol‐generating procedures. On the other hand, if N95 respirators are clearly more effective, then their use for non–aerosol‐generating procedures should be universally recommended. We conducted an updated systematic review and meta‐analysis to help answer this question.

## 2 METHODS

We adhered to the PRISMA statement when reporting of this review (Appendix S1).^14^

### 2.1 Data sources and searches

We adapted search strategies published by Smith et al by removing terms related to surrogate exposure studies (ie, simulations and experiments involving manikins) and applying database‐specific randomized controlled trial (RCT) filters (Appendix S2A‐C).^12^, ^15^ We searched MEDLINE (OVID interface, Epub Ahead of Print, In‐Process & Other Non‐Indexed Citations, 1946 to Present), Embase (OVID interface, 1974 to Present) and the Cochrane Central Register of Controlled Trials (CENTRAL) from January 1, 2014, to March 9, 2020, for English‐language studies to update their search completed on December 9, 2014.^12^ Two reviewers independently and in duplicate screened titles, abstracts and full‐texts of records identified by our searches. Consensus was reached through discussion among the review pair when necessary.

### 2.2 Study selection

We included RCTs that met all the following criteria: the design was an RCT including cluster randomized trials; the intervention was medical masks (defined surgical, procedural, isolation, laser, fluid resistant or face masks certified for use as a medical device) compared with N95 respirators (defined as respirators were N95 filtering face piece respirators certified by the National Institute for Occupational Safety and Health (NIOSH) and European standard filtering facepiece (FFP2) respirators)^12^; the population was healthcare workers (defined as workers in a healthcare setting that could be exposed to a patient with acute respiratory illness) and reported on any of the following outcomes: viral respiratory infection laboratory confirmed by PCR, serology, or viral culture (our primary outcome), laboratory‐confirmed coronavirus infection, laboratory‐confirmed influenza infection, influenza‐like illness, clinical respiratory illness, or workplace absenteeism.

### 2.3 Data extraction and quality assessment

A single reviewer extracted data on study characteristics, participant characteristics, and cases of respiratory illness or infection into a standardized form. A second reviewer completed quality control on the extracted data to ensure its integrity. We combined data from fit tested and non‐fit tested N95 respirator groups for MacIntyre 2011 to generate a single comparator N95 group.^16^ Similarly, we combined targeted use of N95 respirators with non‐targeted use of N95 respirators for MacIntrye 2013.^17^ For Radonovich 2019, we used healthcare worker‐seasons as the population metric (denominator) given that healthcare workers were treated independently and allowed to participate for up to all 4 years the study was conducted (2011/12 to 2014/15).^18^

Reviewers assessed risk of bias of eligible RCTs independently and in duplicate using a modified Cochrane risk of bias tool.^19^ Selection bias (random sequence generation and allocation concealment), performance bias (blinding of participants and personnel and other threats to validity), detection bias (blinding of outcome assessment and other potential threats to validity), attrition bias (incomplete outcome data), and reporting bias (selective outcome reporting assessed by comparing outcomes reported in the protocol to those in the published study or by comparing outcomes reported in the results to those in the methods of the published study) were assessed. For each domain in the tool, trials judged as definitely or probably being free of a given risk of bias were considered low risk of bias, whereas trials judged as probably or definitely biased were considered high risk of bias to reduce reporting of unclear bias assessments. For each outcome, we considered individual trials to be at serious risk of bias overall if 2 of the 8 risk of bias domains were judged as high risk and very serious risk of bias overall if more than 2 domains were judged as high risk. Similarly, reviewers assessed the certainty in the evidence independently and in duplicate using the *grading of recommendations assessment, development and evaluation* (GRADE) approach.^20^ The certainty we can have in our evidence ranges from very low, low, and moderate to high. It depends on risk of bias, inconsistency, indirectness, imprecision, and other considerations like publication bias. The Cochrane risk of bias tool and GRADE were applied at the outcome level. Consensus was reached through discussion among the review pair or with consultation of a third reviewer when necessary.

### 2.4 Data synthesis and analysis

Pooled odds ratios (ORs) and corresponding 95% confidence intervals (CIs) comparing medical masks to N95 respirators on dichotomous outcomes were calculated in R Project for Statistical Computing (version 3.6.3). The “metafor” package was used, applying the inverse variance method and assuming a random effects model due to expected heterogeneity between studies.^21^, ^22^ We set the criterion for statistical significance at alpha = 0.05. Visual inspection of forest plots and the chi‐square test were performed to evaluate heterogeneity. An I^2^ statistic value of 0%‐40%, 30%‐60%, 50%‐90%, or 75%‐100% was interpreted as not likely important, moderate, substantial, or considerable heterogeneity, respectively.^23^ When inconsistent magnitudes and directions of effect were observed upon visual inspection of the forest plot, and the chi‐square test was significant, we interpreted heterogeneity as more important (ie, interpretation corresponding to the higher range in overlapping *I* ^2^ statistic values was reported).^23^

To avoid unit‐of‐analysis errors in pooling data from cluster‐RCTs with individual participant RCTs, we adjusted meta‐analyses by calculating the effective sample sizes of included cluster‐RCTs. We used data on the average cluster size and intraclass correlation coefficient (ICC) to calculate the design effect of the cluster‐RCT when not reported. Individual level data were divided by the design effect to calculate the effective sample sizes (ie, number of events in each trial arm and the total sample size of each trial arm were reduced by the amount of correlation in clusters). We rounded effective sample sizes to the nearest whole number to be meta‐analyzed.^24^ Aggregate data from the trials, corresponding effective sample sizes, and the statistical parameters used to calculate the effective sample sizes are available upon request.

### 2.5 Role of the funding source

This study was conducted without financial support.

## 3 RESULTS

### 3.1 Search results and study characteristics

**Figure 1:**
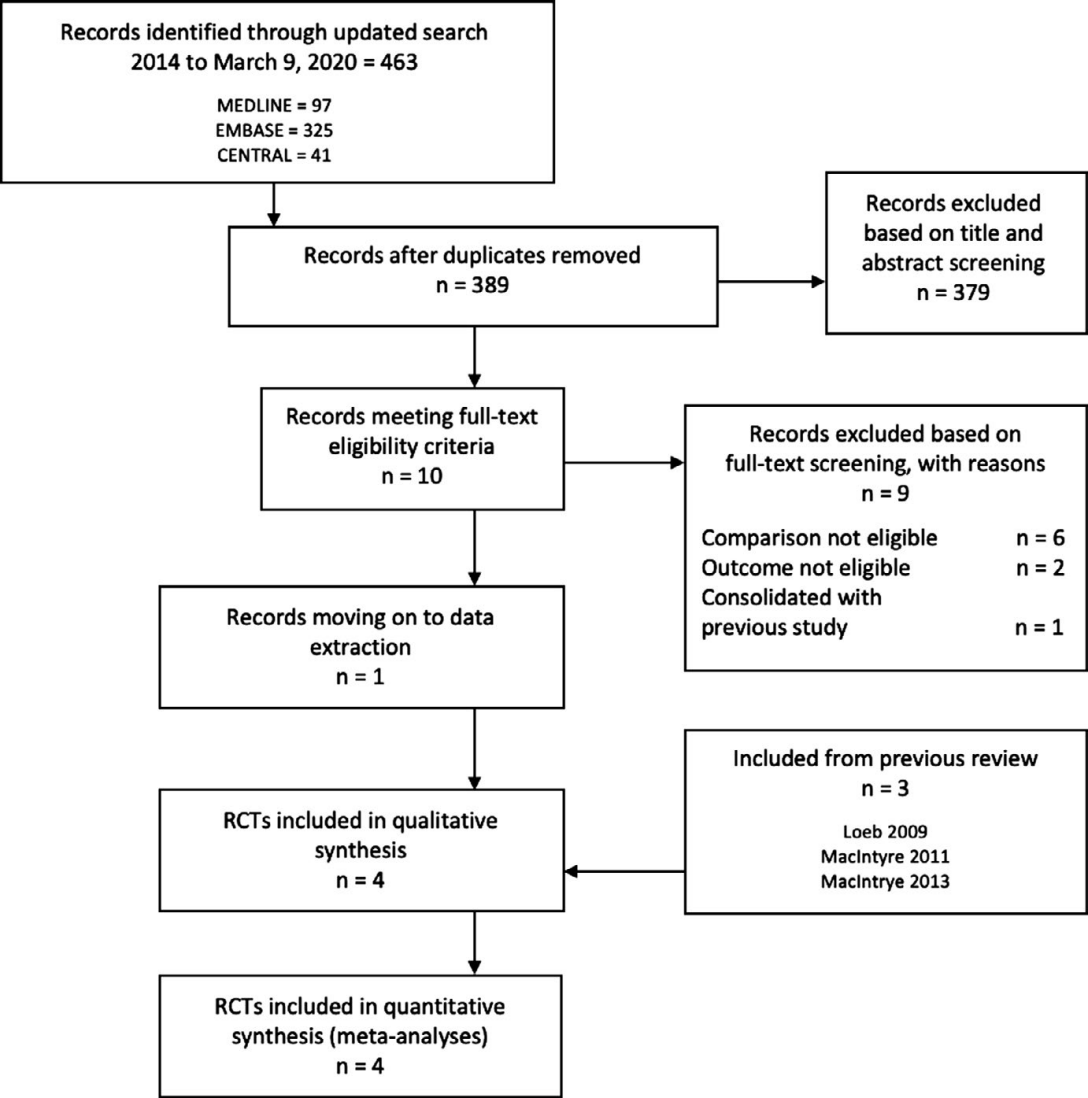
PRISMA study flow diagram (randomized controlled trials, RCTs)

Our systematic review update identified 1 new randomized trial (n = 5180) eligible for meta‐analysis following screening of 389 titles and abstracts, and 12 full‐texts (Figure 1).^18^ To date, there have been four trials where healthcare workers providing care for patients with acute febrile illness were randomized to medical masks (n = 3957) or N95 respirators (n = 4779), of which 3 were identified from a 2016 systematic review (Table 1).^16^, ^17^, ^18^, ^25^ Three of the trials were cluster‐randomized,^16^, ^17^, ^18^ and one was not.^25^ Two trials were conducted in North America (Canada and US),^18^, ^25^ and two were conducted in China.^16^, ^17^ All randomized trials reported on laboratory‐confirmed viral respiratory infection, defined by the detection of viral RNA using reverse‐transcriptase PCR from nasopharyngeal and flocked nasal specimens.^16^, ^17^, ^18^, ^25^ All studies included PCR testing for respiratory viruses in the *Coronavidiae* family^16^, ^17^, ^18^, ^25^; however, only one trial reported results directly on coronavirus (OC43, 229E, SARS, NL63, and HKU1) infection.^25^ Laboratory‐confirmed influenza infection (using PCR or hemagglutination inhibition) and influenza‐like illness (based on pre‐determined respiratory symptoms and fever ≥ 38˚C) were also studied in all 4 randomized trials.^16^, ^17^, ^18^, ^25^ The criteria for clinical respiratory illness varied among trials and are detailed in the Appendix S3.^16^, ^17^, ^18^ Only Loeb 2009 reported on workplace absenteeism.^25^

**Table 1:** Characteristics of the studies included in meta‐analysis

Study	Setting	Healthcare Workers	Viral Testing	Outcomes
Loeb (2009)^25^	Emergency departments, medical units and pediatric units; 8 tertiary care hospitals in Ontario (6 in Toronto); Canada	446 nurses during the 2008‐2009 influenza season in routine care, individually randomized	Influenza A and B; Non‐influenza viruses: parainfluenza virus types 1, 2, 3, and 4; respiratory syncytial virus types A and B; adenovirus; metapneumovirus; rhinovirus‐enterovirus; and coronaviruses OC43, 229E, SARS, NL63, and HKU1	Primary:laboratory‐confirmed influenza; Secondary: respiratory syncytial virus; metapneumovirus; parainfluenza virus; rhinovirus‐enterovirus; coronavirus; laboratory‐confirmed viral respiratory infection; influenza‐like illness; work‐related absenteeism
MacIntyre (2011)^16^	Emergency departments and respiratory wards; 15 hospitals in Beijing; China	1441 nurses, doctors and ward clerks cluster‐randomized by hospital during the winter season (December 2008 to January 2009); 33% participating in high‐risk procedures^a^	Adenoviruses, human metapneumovirus, coronavirus 229E ⁄ NL63, parainfluenza viruses 1, 2 and 3, influenza A and B, respiratory syncytial virus A and B, rhinovirus A⁄ B and coronavirus OC43 ⁄HKU1	Primary: laboratory‐confirmed viral respiratory infection; influenza infection; influenza‐like illness; clinical respiratory illness
MacIntyre (2013)^17^	68 wards (emergency departments and respiratory wards); 19 tertiary hospitals in Beijing; China	1669 nurses and doctors cluster‐randomized by ward during the winter season (December 2009 to February 2010); 73% undertook high‐risk procedures^a^	Adenoviruses; human metapneumovirus; coronaviruses 229E/NL63 and OC43/HKU1; parainfluenza viruses 1, 2, and 3; influenza A and B; respiratory syncytial viruses A and B; or rhinoviruses A/B	Primary: laboratory‐confirmed viral respiratory infection; laboratory‐confirmed influenza infection; influenza‐like illness, clinical respiratory illness
Radonovich (2019)^18^	137 study sites comprised of varying outpatient settings: primary care facilities, dental clinics, adult and pediatric clinics, dialysis units, urgent care facilities and emergency departments, and emergency transport services; across 7 medical centers; USA	2862 healthcare personnel cluster‐randomized by study site during 4 viral respiratory seasons (2011/12 to 2014/15); 60% at occupational high risk^a^	Coxsackie/echoviruses; coronaviruses HKU1, NL63, OC43, and 229E; human metapneumovirus; human rhinovirus; influenza A and B; parainfluenza virus types 1, 2, 3, and 4; respiratory syncytial virus types A and B	Primary: laboratory‐confirmed influenza infection; Secondary: laboratory‐confirmed viral respiratory infection; influenza‐like illness; clinical respiratory illness

^a^ High risk consisted of physical examination, barrier nursing of a patient with known respiratory illness, intubation, airway suctioning, nebulizer treatments, nasopharyngeal aspiration, aerosol‐generating procedures, and/or chest physiotherapy.

### 3.2 Effect on outcomes

**Figure 2:**
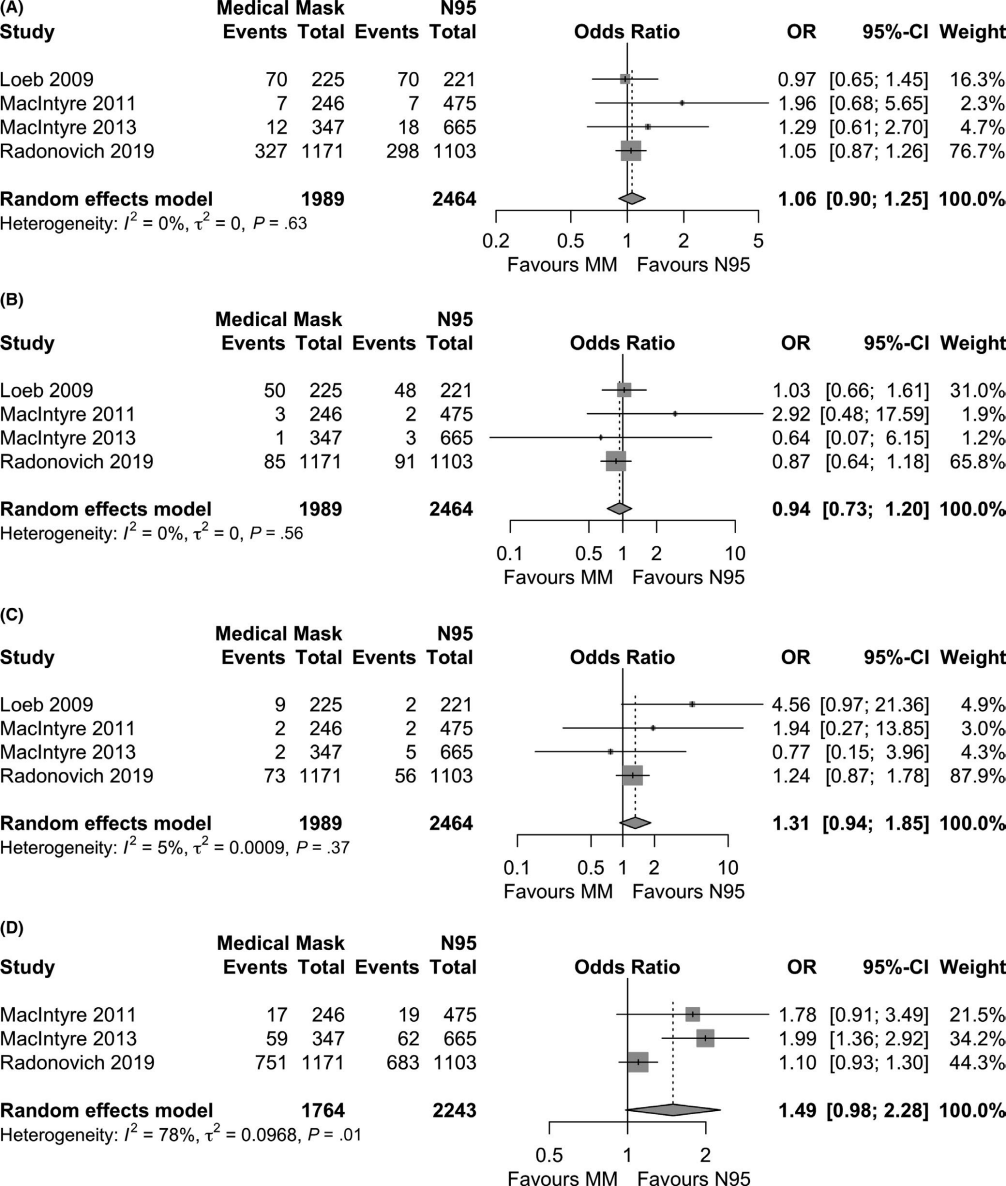
Meta‐analyses of 4 randomized controlled trials comparing medical masks to N95 respirators in preventing A, Laboratoryconfirmed viral respiratory infection; B, Laboratory‐confirmed influenza infection; C, Influenza‐like illness; and D, Clinical respiratory illness

For laboratory‐confirmed viral respiratory infection, the pooled effect of medical masks compared to N95 respirators was OR 1.06 (95% CI 0.90‐1.25), *I* ^2^ = 0% (Figure 2A). For laboratory‐confirmed influenza infection, OR 0.94 (95% CI: 0.73‐1.20), *I* ^2^ = 0% (Figure 2B). For influenza‐like illness, the effect was OR 1.31 (95% CI: 0.94‐1.85), *I* ^2^ = 5% (Figure 2C).^16^, ^17^, ^18^, ^25^ For clinical respiratory illness, OR 1.49 (95% CI: 0.98‐2.28), *I* ^2^ = 78% (Figure 2D). ^16^, ^17^, ^18^ Since only one trial reported directly on coronavirus and workplace absenteeism; meta‐analysis was not possible.^25^ When seasonal coronavirus (OC43, HKU1, 229E, NL63) was tested for by PCR in this non‐cluster randomized trial of medical masks vs N95 respirators, 4.3% (9/212) of nurses in the medical mask group had RT‐PCR confirmed coronavirus infection compared with 5.7% (12/210) in the N95 respirator group (*P* = .49). Work‐related absenteeism was reported in 19.8% (42/212) of nurses in the medical mask group compared with 18.6% (39/210) of nurses in the N95 respirator group (*P* = .75).^25^

### 3.3 Quality assessment

Using the Cochrane risk of bias tool, randomized trials were judged to have low risk of selection, attrition and reporting biases. Risk of performance bias was high across all trials and outcomes due to lack of blinding of participant healthcare workers. Risk of detection bias was high for influenza‐like and clinical respiratory illness due to lack of laboratory confirmation, but low for laboratory‐confirmed viral respiratory and influenza infection (Appendix S4). In applying GRADE, the certainty of the overall evidence was judged to be low for laboratory‐confirmed respiratory infection and laboratory‐confirmed influenza infection, and very low for influenza‐like illness and clinical respiratory illness, largely due to indirectness and imprecision (Table 2).

**Table 2:** Evidence profile of 4 randomized controlled trials synthesized quantitatively

Question: Medical Masks compared to N95 Respirators for COVID‐19												
Setting: Healthcare workers; Outpatient settings and hospitals; Canada, US, China												
Certainty assessment							№ of patients		Effect		Certainty	Importance
№ of studies	Study design	Risk of bias	Inconsistency	Indirectness	Imprecision	Other considerations	Medical Masks	N95 Respirators	Relative (95% CI)	Absolute (95% CI)		
Laboratory‐confirmed respiratory viral infection												
4	Randomized trials	Not serious^a^	Not serious	Serious^b^	Serious^c^	None	416/1989 (20.9%)	393/2464 (15.9%)	OR 1.06 (0.90‐1.25)	8 more per 1000 (from 14 fewer to 32 more)	⨁⨁◯◯ LOW	Critical
Laboratory‐confirmed influenza infection												
4	Randomized trials	Not serious^a^	Not serious	Serious^b^	Serious^c^	None	139/1989 (7.0%)	144/2464 (5.8%)	OR 0.94 (0.73‐1.20)	3 fewer per 1000 (from 15 fewer to 11 more)	⨁⨁◯◯ LOW	Important
Influenza‐like illness												
4	Randomized trials	Serious^d^	Not serious	Serious^b^	Serious^c^	None	86/1989 (4.3%)	65/2464 (2.6%)	OR 1.31 (0.94‐1.85)	8 more per 1000 (from 2 fewer to 21 more)	⨁◯◯◯ VERY LOW	Important
Clinical respiratory illness												
3	Randomized trials	Serious^d^	Very serious^e^	Serious^b^	Very serious^f^	None	827/1764 (46.9%)	764/2243 (34.1%)	OR 1.49 (0.98‐2.28)	94 more per 1000 (from 5 fewer to 200 more)	⨁◯◯◯ VERY LOW	Important

Abbreviations: CI, Confidence interval; OR, Odds ratio.

^a^ Lack of blinding of participants.

^b^ Studies were not specific to coronaviruses.

^c^ Wide confidence intervals of individual and pooled estimates.

^d^ Lack of blinding of participants and laboratory confirmation.

^e^ I^2^ 78%, significant test for heterogeneity, difference in magnitude of effect between 1 large and 2 small studies.

^f^ Very wide confidence intervals of individual and pooled studies.

## 4 DISCUSSION

There is no convincing evidence that medical masks are inferior to N95 respirators for protecting healthcare workers against laboratory‐confirmed viral respiratory infections during routine care and non–aerosol‐generating procedures. Medical masks also performed similarly to N95 respirators in preventing laboratory‐confirmed influenza infection. For influenza‐like illnesses and clinical respiratory illnesses, the point estimates favored N95 respirators; however, the confidence intervals were wide and there was considerable heterogeneity for the clinical respiratory illness outcome (*P* = .01, *I* ^2^ = 78%). This heterogeneity may have been due to the subjective nature of the criteria used to define this outcome between trials (Appendix S3). Reduced protection with medical masks during routine care of COVID‐19 patients cannot be ruled out. Our low certainty in available evidence is because of its indirectness. When we searched for randomized trials comparing the protective effect of medical masks to N95 respirators against coronaviruses, we did not identify any for novel SARS‐CoV‐2 causing COVID‐19.

Our findings support preliminary epidemiological data from a case‐report of respiratory protective devices for COVID‐19.^26^ Forty‐one healthcare workers were exposed to aerosol‐generating procedures from a patient with severe pneumonia, who later tested positive for SARS‐CoV‐2 during COVID‐19 surveillance. These procedures included endotracheal intubation, extubation, non‐invasive ventilation, and exposure to aerosols in an open circuit. All of the exposed healthcare workers tested negative 14 days after their date of exposure, despite 85% (35/41) having worn surgical masks during the high‐risk procedures.^26^ Given the limited direct evidence from this case‐report, further research on the risk of secondary infection in healthcare workers caring for COVID‐19 patients is warranted.

There are several limitations in this meta‐analysis. First, only one trial individually studied cases of coronavirus infection between medical masks and N95 respirators; therefore, we were unable to meta‐analyze coronavirus infection specifically. This led us to downgrade the evidence with GRADE as it relates to indirectness because our findings may not be generalizable to SARS‐CoV‐2. All trials however did report a composite outcome of laboratory‐confirmed viral respiratory infections that included coronavirus infections. Second, this is a meta‐analysis of aggregate data, rather than individual data. The latter would allow for harmonization of confounding co‐variates and outcome definitions, specific to coronavirus infection. The definition of influenza‐like illness was based on a pre‐determined set of signs and symptoms; however, swabs in these studies were likely obtained using a more lenient threshold. Therefore, there would have been swabs obtained even when the influenza‐like illness criteria were not met. This likely resulted in fewer influenza‐like illness events compared with laboratory‐confirmed influenza infections. Reassuringly, outcome definitions (excluding clinical respiratory illness) were consistent among all four studies.

A strength of this review is that it is up‐to‐date and incorporates the largest randomized trial of medical masks vs N95 respirators that have been completed to date.^18^ Secondly, we used appropriate meta‐analytic techniques that accounted for cluster randomization that was present in three of the four included trials.^16^, ^17^, ^18^ Cluster randomization was not adjusted for in the most recent meta‐analysis potentially leading to falsely narrower confidence intervals around point estimates of protection.^13^, ^27^

For aerosol‐generating procedures, N95 respirators are unanimously recommended by national and international guidelines; however, there is inconsistency in recommendations for routine care and non–aerosol‐generating procedures of COVID‐19 patients.^6^, ^7^, ^8^, ^9^ Our evidence is in keeping with current WHO and Public Health Agency of Canada recommendations to use medical masks for non–aerosol‐generating procedures when caring for COVID‐19 patients. In contrast, the CDC and ECDC recommend use of N95 respirators for non–aerosol‐generating procedures over the less expensive and more readily available medical masks.

With the widespread of SARS‐CoV‐2, a serious concern is that stockpiles of N95 respirators will be depleted. The Department of Health and Human Services announced that its Strategic National Stockpile—the emergency stockpile of drugs and medical supplies in the United States—contained approximately 30 million medical masks and 12 million N95 respirators. This stockpile of respiratory protective devices equates to 1% of the estimated amount needed for U.S HCWs in a pandemic scenario (42 million stockpiled compared with the estimated 3.5 billion needed).^28^ Based on the evidence, preservation of N95 respirators for high‐risk, aerosol‐generating procedures in this pandemic should be considered when in short supply. The uncertainty of this evidence and the depleting stockpiles of respiratory protective devices emphasize the need for further comparative research of medical masks and N95 respirators.

## AUTHOR CONTRIBUTIONS

**Jessica J. Bartoszko: **Data curation‐Equal, Formal analysis‐Lead, Methodology‐Lead, Project administration‐Lead, Software‐Equal, Visualization‐Equal, Writing‐original draft‐Equal, Writing‐review & editing‐Equal. **Mohammed Abdul Malik Farooqi: **Data curation‐Equal, Formal analysis‐Supporting, Visualization‐Equal, Writing‐original draft‐Equal, Writing‐review & editing‐Equal. **Waleed Alhazzani: **Conceptualization‐Equal, Investigation‐Equal, Supervision‐Equal, Writing‐review & editing‐Equal. **Mark Loeb: **Conceptualization‐Equal, Investigation‐Equal, Supervision‐Equal, Writing‐review & editing‐Lead.

## Supporting information

Appendix S1‐S4
