# Supplementary material for: Medical masks vs N95 respirators for preventing COVID‐19 in healthcare workers: A systematic review and meta‐analysis of randomized trials
Source: Influenza Other Respir Viruses. 2020 Apr 21;14(4):365–73. doi: 10.1111/irv.12745 (PMC7298295; doi:10.1111/irv.12745)
Supplement: Supplementary file 1 — Appendix S1‐S4 [file IRV-14-365-s001.docx]

**Appendix S1.** PRISMA checklist.

| **Section/topic** | **#** | **Checklist item** | **Reported on page #** |
| --- | --- | --- | --- |
| **TITLE** | | |  |
| Title | 1 | Identify the report as a systematic review, meta-analysis, or both. | 1 |
| **ABSTRACT** | | |  |
| Structured summary | 2 | Provide a structured summary including, as applicable: background; objectives; data sources; study eligibility criteria, participants, and interventions; study appraisal and synthesis methods; results; limitations; conclusions and implications of key findings; systematic review registration number. | 2 |
| **INTRODUCTION** | | |  |
| Rationale | 3 | Describe the rationale for the review in the context of what is already known. | 4-5 |
| Objectives | 4 | Provide an explicit statement of questions being addressed with reference to participants, interventions, comparisons, outcomes, and study design (PICOS). | 4-5 |
| **METHODS** | | |  |
| Protocol and registration | 5 | Indicate if a review protocol exists, if and where it can be accessed (e.g., Web address), and, if available, provide registration information including registration number. | NA |
| Eligibility criteria | 6 | Specify study characteristics (e.g., PICOS, length of follow-up) and report characteristics (e.g., years considered, language, publication status) used as criteria for eligibility, giving rationale. | 6-9 |
| Information sources | 7 | Describe all information sources (e.g., databases with dates of coverage, contact with study authors to identify additional studies) in the search and date last searched. | 6-9 |
| Search | 8 | Present full electronic search strategy for at least one database, including any limits used, such that it could be repeated. | 6-9 |
| Study selection | 9 | State the process for selecting studies (i.e., screening, eligibility, included in systematic review, and, if applicable, included in the meta-analysis). | 6-9 |
| Data collection process | 10 | Describe method of data extraction from reports (e.g., piloted forms, independently, in duplicate) and any processes for obtaining and confirming data from investigators. | 6-9 |
| Data items | 11 | List and define all variables for which data were sought (e.g., PICOS, funding sources) and any assumptions and simplifications made. | 6-9 |
| Risk of bias in individual studies | 12 | Describe methods used for assessing risk of bias of individual studies (including specification of whether this was done at the study or outcome level), and how this information is to be used in any data synthesis. | 6-9 |
| Summary measures | 13 | State the principal summary measures (e.g., risk ratio, difference in means). | 6-9 |
| Synthesis of results | 14 | Describe the methods of handling data and combining results of studies, if done, including measures of consistency (e.g., I^2^) for each meta-analysis. | 6-9 |

Page 1 of 2

| **Section/topic** | **#** | **Checklist item** | **Reported on page #** |
| --- | --- | --- | --- |
| Risk of bias across studies | 15 | Specify any assessment of risk of bias that may affect the cumulative evidence (e.g., publication bias, selective reporting within studies). | 6-9 |
| Additional analyses | 16 | Describe methods of additional analyses (e.g., sensitivity or subgroup analyses, meta-regression), if done, indicating which were pre-specified. | 6-9 |
| **RESULTS** | | |  |
| Study selection | 17 | Give numbers of studies screened, assessed for eligibility, and included in the review, with reasons for exclusions at each stage, ideally with a flow diagram. | 9-11 |
| Study characteristics | 18 | For each study, present characteristics for which data were extracted (e.g., study size, PICOS, follow-up period) and provide the citations. | 9-11 |
| Risk of bias within studies | 19 | Present data on risk of bias of each study and, if available, any outcome level assessment (see item 12). | 9-11 |
| Results of individual studies | 20 | For all outcomes considered (benefits or harms), present, for each study: (a) simple summary data for each intervention group (b) effect estimates and confidence intervals, ideally with a forest plot. | 9-11 |
| Synthesis of results | 21 | Present results of each meta-analysis done, including confidence intervals and measures of consistency. | 9-11 |
| Risk of bias across studies | 22 | Present results of any assessment of risk of bias across studies (see Item 15). | 9-11 |
| Additional analysis | 23 | Give results of additional analyses, if done (e.g., sensitivity or subgroup analyses, meta-regression [see Item 16]). | 9-11 |
| **DISCUSSION** | | |  |
| Summary of evidence | 24 | Summarize the main findings including the strength of evidence for each main outcome; consider their relevance to key groups (e.g., healthcare providers, users, and policy makers). | 11-14 |
| Limitations | 25 | Discuss limitations at study and outcome level (e.g., risk of bias), and at review-level (e.g., incomplete retrieval of identified research, reporting bias). | 11-14 |
| Conclusions | 26 | Provide a general interpretation of the results in the context of other evidence, and implications for future research. | 11-14 |
| **FUNDING** | | |  |
| Funding | 27 | Describe sources of funding for the systematic review and other support (e.g., supply of data); role of funders for the systematic review. | 9 |

**Appendix S2.** Search Strategies.

**A)** Database: OVID Medline Epub Ahead of Print, In-Process & Other Non-Indexed Citations, Ovid MEDLINE(R) Daily and Ovid MEDLINE(R) 1946 to Present

***Note***

Have removed the following lines related to surrogate studies from the published search

13 Manikins/ or "Nebulizers and Vaporizers"/

14 ("breathing simulator" or "cascade impaction" or (air adj sampler$) or "head form$" or headform$ or

"head-form$" or airtight or chamber$1 or hood$1 or man?equin$ or man?ikin$ or nebulizer$ or

simulat$ or surrogate$ or human$ or volunteer$ or subject$).ti,ab,kf.

15 13 or 14

Search Strategy:

--------------------------------------------------------------------------------

1 Masks/ or Protective Devices/ or Respiratory Protective Devices/ (12586)

2 (masks or mask or facemask$ or respirator or respirators or N95 or FFP2 or "personal protection equipment" or "personal protective equipment").ti,ab,kf. (43802)

3 1 or 2 (51776)

4 Aerosols/ or Particle Size/ or Particulate Matter/ or Adenovirus Infections, Human/ or Adenoviruses, Human/ or Bacteria/ or Bacterial Infections/ or Bordetella Infections/ or Bordetella pertussis/ or Chickenpox/ or Communicable Diseases, Emerging/ or Communicable Diseases/ or Coronavirus Infections/ or Coronavirus/ or Disease Outbreaks/ or exp Cross Infection/ or exp Disease Transmission, Infectious/ or exp Haemophilus influenzae/ or exp Orthomyxoviridae/ or exp Pneumonia, Bacterial/ or exp Pneumovirus Infections/ or exp Respiratory Tract Infections/ or exp Respirovirus Infections/ or Gram-Negative Bacterial Infections/ or Herpesvirus 3, Human/ or Infection Control/ or Infection/ or Influenza, Human/ or Metapneumovirus/ or Paramyxoviridae Infections/ or Pneumonia, Viral/ or Pneumonia/ or Respiratory Syncytial Virus Infections/ or Respiratory Syncytial Virus, Human/ or Respiratory Syncytial Viruses/ or Respiratory Tract Diseases/ or Rhinovirus/ or SARS Virus/ or Severe Acute Respiratory Syndrome/ or transmission.fs. or Virus Diseases/ or Viruses/ or Whooping Cough/ (1112748)

5 (particle$ or "particulate matter" or aerosol$ or bioaerosol$ or (acute adj2 respiratory) or adenovirus$ or airborne$ or ARI or bacteri$ or chickenpox or "chicken pox" or communicable$ or coronavirus or CRI or cross infect$ or disease$ or droplet$ or (emerg$ adj2 pathogen$) or epidemic$ or flu or H1N1 or haemophilus or "health care acquired" or "health care associated" or "healthcare acquired" or "healthcare associated" or "hospital acquired" or "hospital associated" or HiB or ILI or illness$ or incidence or infect$ or influenza$ or measles or MERS or metapneumovirus$ or "Middle East respiratory syndrome" or nosocomial or orthomyxoviridae or outbreak$ or pandemic$ or parainfluenza or paramyxoviridae or particle$ or pathogen$ or pneumonia$ or (respiratory adj2 disease$) or (respiratory adj2 illness$) or (respiratory adj2 infection$) or "respiratory hygiene" or ("respiratory syncytial" adj1 virus$) or "respiratory tract" or rhinovirus or RSV or SARS or "severe acute respiratory syndrome" or sick$ or syncytial or transmission or varicella or viral or virion$ or virus$ or pertussis or "whooping cough").ti,ab,kf. (7489289)

6 4 or 5 (7790218)

7 Filtration/ or exp Occupational Exposure/ or Inhalation Exposure/ or exp Microbial Viability/ (105165)

8 ("face seal" or faceseal or face-seal or filter$ or filtrat$ or leak$ or penetrat$ or "viral viability" or "viral culture" or "bacterial culture" or WPF or APF or SWPF or "protection factor" or "5th percentile" or "bacteria$ viability" or "virus viability" or "virus culture").ti,ab,kf. (507314)

9 7 or 8 (592912)

10 exp Health Personnel/ (503777)

11 (doctor$ or physician$ or clinician$ or nurse$ or nursing or paramedic$ or ((health$ or hospital$ or nurs$ or clinical or care or medical$) adj2 (practitioner$ or staff or personnel or worker$ or employee$ or provider$ or professional$)) or HCW or HCP).ti,ab,kf. (1305728)

12 10 or 11 (1560801)

13 3 and (6 or 9) and 12 (3234)

14 limit 13 to english language (2912)

15 limit 14 to yr="2014 -Current" (1069)

16 randomized controlled trial.pt. (501215)

17 (random$ or placebo$ or single blind$ or double blind$ or triple blind$).ti,ab. (1208253)

18 (retraction of publication or retracted publication).pt. (14983)

19 or/16-18 (1320951)

20 (animals not humans).sh. (4642514)

21 ((comment or editorial or meta-analysis or practice-guideline or review or letter) not randomized controlled trial).pt. (4470796)

22 (random sampl$ or random digit$ or random effect$ or random survey or random regression).ti,ab. not randomized controlled trial.pt. (86914)

23 19 not (20 or 21 or 22) (967775)

24 15 and 23 (97)

***************************

**B)** Database: Embase <1974 to 2020 March 05>

***Note***

Have removed the following lines related to surrogate studies from the published search

13 simulation/ or exp nebulizer/

14 ("breathing simulator" or "cascade impaction" or (air adj sampler$) or "head form$" or headform$ or

"head-form$" or airtight or chamber$1 or hood$1 or man?equin$ or man?ikin$ or nebulizer$ or

simulat$ or surrogate$ or human$ or volunteer$ or subject$).ti,ab,kw.

15 13 or 14

Search Strategy:

--------------------------------------------------------------------------------

1 exp mask/ or face mask/ or surgical mask/ or protective equipment/ or ventilator/ (65702)

2 (masks or mask or facemask$ or respirator or respirators or N95 or FFP2 or "personal protection equipment" or "personal protective equipment").ti,ab,kw. (56377)

3 1 or 2 (103585)

4 aerosol/ or particle size/ or particulate matter/ or human adenovirus infection/ or human adenovirus/ or bacterium/ or bacterial infection/ or chickenpox/ or communicable disease/ or Coronavirus infection/ or Coronavirus/ or SARS coronavirus/ or cross infection/ or hospital infection/ or airborne infection/ or healthcare associated infection/ or epidemic/ or pandemic/ or disease transmission/ or virus transmission/ or bacterial transmission/ or exp Haemophilus influenzae/ or exp Orthomyxovirus/ or bacterial pneumonia/ or pneumonia/ or virus pneumonia/ or exp infectious pneumonia/ or severe acute respiratory syndrome/ or exp Pneumovirus infection/ or exp respiratory tract infection/ or respiratory tract disease/ or exp Respirovirus infection/ or Rhinovirus infection/ or exp Human rhinovirus/ or Rhinovirus/ or Respiratory syncytial pneumovirus/ or respiratory syncytial virus infection/ or exp influenza/ or infection/ or infection control/ or infection prevention/ or infection risk/ or paramyxovirus infection/ or virus infection/ or virus/ or Varicella zoster virus/ or metapneumovirus/ or human metapneumovirus/ or Gram negative infection/ or pertussis/ or Bordetella pertussis/ or bordetellosis/ (1826776)

5 (particle$ or "particulate matter" or aerosol$ or bioaerosol$ or (acute adj2 respiratory) or adenovirus$ or airborne$ or ARI or bacteri$ or chickenpox or "chicken pox" or communicable$ or coronavirus or CRI or cross infect$ or disease$ or droplet$ or (emerg$ adj2 pathogen$) or epidemic$ or flu or H1N1 or haemophilus or "health care acquired" or "health care associated" or "healthcare acquired" or "healthcare associated" or "hospital acquired" or "hospital associated" or HiB or ILI or illness$ or incidence or infect$ or influenza$ or measles or MERS or metapneumovirus$ or "Middle East respiratory syndrome" or nosocomial or orthomyxoviridae or outbreak$ or pandemic$ or parainfluenza or paramyxoviridae or particle$ or pathogen$ or pneumonia$ or (respiratory adj2 disease$) or (respiratory adj2 illness$) or (respiratory adj2 infection$) or "respiratory hygiene" or ("respiratory syncytial" adj1 virus$) or "respiratory tract" or rhinovirus or RSV or SARS or "severe acute respiratory syndrome" or sick$ or syncytial or transmission or varicella or viral or virion$ or virus$ or pertussis or "whooping cough").ti,ab,kw. (9335282)

6 4 or 5 (9737381)

7 filtration/ or occupational exposure/ or exposure/ or exp microbial viability/ (295736)

8 ("face seal" or faceseal or face-seal or filter$ or filtrat$ or leak$ or penetrat$ or "viral viability" or "viral culture" or "bacterial culture" or WPF or APF or SWPF or "protection factor" or "5th percentile" or "bacteria$ viability" or "virus viability" or "virus culture").ti,ab,kw. (642538)

9 7 or 8 (900101)

10 exp health care personnel/ (1505826)

11 (doctor$ or physician$ or clinician$ or nurse$ or nursing or paramedic$ or ((health$ or hospital$ or nurs$ or clinical or care or medical$) adj2 (practitioner$ or staff or personnel or worker$ or employee$ or provider$ or professional$)) or HCW or HCP).ti,ab,kw. (1666286)

12 10 or 11 (2513584)

13 3 and (6 or 9) and 12 (9764)

14 limit 13 to english language (9235)

15 limit 14 to yr="2014 -Current" (4603)

16 (random$ or placebo$ or single blind$ or double blind$ or triple blind$).ti,ab. (1641344)

17 RETRACTED ARTICLE/ (9268)

18 or/16-17 (1650201)

19 (animal$ not human$).sh,hw. (4328382)

20 (book or conference paper or editorial or letter or review).pt. not exp randomized controlled trial/ (5015461)

21 (random sampl$ or random digit$ or random effect$ or random survey or random regression).ti,ab. not exp randomized controlled trial/ (110688)

22 18 not (19 or 20 or 21) (1262921)

23 15 and 22 (325)

***************************

**C)** Database: CENTRAL

***Note***

Have removed the following lines related to surrogate studies from the published search

S5 "breathing simulator" or "cascade impaction" or (air N3 sampler*) or "head form*" or headform* or

"head-form*" or airtight or chamber* or hood* or man#equin* or man#ikin* or nebulizer* or simulat*

or surrogate* or human* or volunteer* or subject*

Search strategy:


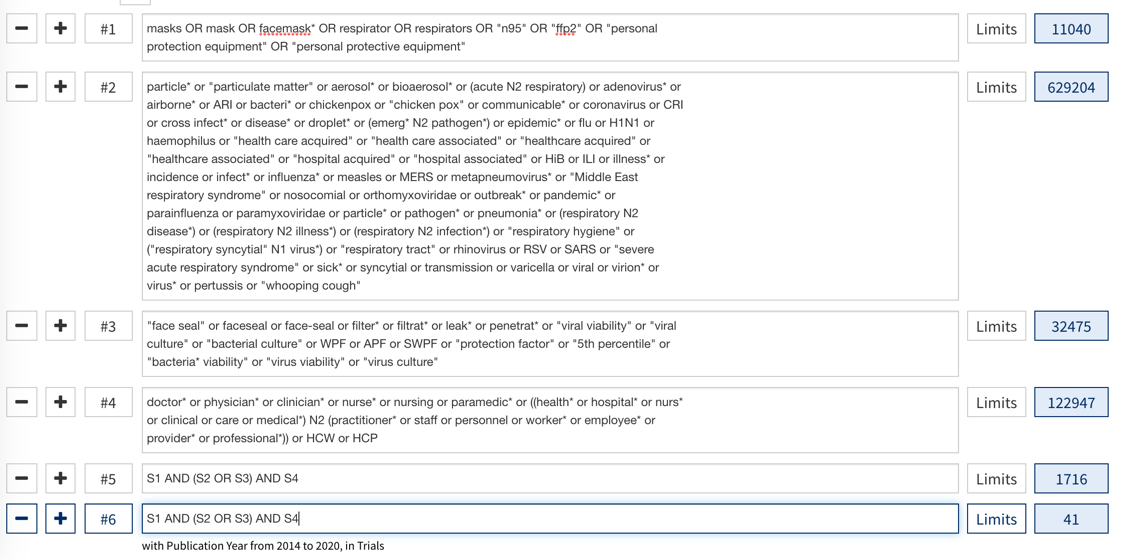


**Appendix S3.** Study definitions of clinical respiratory illness.

| Study | Criteria |
| --- | --- |
| MacIntyre 2011, 2013 | - Two or more respiratory symptoms or 1 respiratory symptom and 1 systemic symptom. Examples listed below (not limited to these examples):   - Respiratory symptoms     - Cough     - Runny nose     - Sore throat     - Nasal congestion   - Systemic symptom     - Myalgia     - Arthralgia     - Fever     - Headache |
| Radonovich 2019 | - Presence of at least 1 sign or 2 symptoms limited to the list below:   - Signs     - Coryza     - Fever (temperature >37.8˚C)     - Lymphadenopathy     - Tachypnea (respiratory rate >25 breaths/min)   - Symptoms     - Arthralgias or myalgias     - Chills     - Cough     - Diarrhea     - Dyspnea     - Fatigue     - Headache     - Malaise     - Other gastrointestinal symptoms     - Sore throat     - Sputum production     - Sweats     - Vomiting or nausea |

**Appendix S4.** Cochrane risk of bias tool for randomized controlled trials: risk of bias assessment (n = 4).†

| Author (Year) | Selection bias | | | | Performance bias | | Detection bias | | Attrition bias | | Reporting bias | | Other biases | |
| --- | --- | --- | --- | --- | --- | --- | --- | --- | --- | --- | --- | --- | --- | --- |
|  | Random sequence generation | | Allocation concealment | | Blinding of Participants | | Blinding of outcome assessment | | Loss to follow-up | | Selective outcome reporting? | | Funding? | |
| Loeb (2009) | Low | Central randomization | Low | Central randomization | High | Cannot blind |  | Laboratory personnel blinded; low for laboratory confirmed outcomes and high for respiratory illnesses | Low | <10% and reasons provided | Low | trial registered | Low | Not for-profit |
| MacIntyre (2011) | Low | Central randomization | Low | Central randomization | High | Cannot blind | High | No mention, high for all outcomes | Low | n randomized = n analyzed | Low | trial registered | Low | Not for-profit |
| MacIntyre (2013) | Low | Central randomization | Low | Central randomization | High | Cannot blind | High | No mention, high for all outcomes | Low | n randomized = n analyzed | Low | trial registered |  | NR |
| Radonovich (2019) | Low | Central randomization | Low | Central randomization | High | Cannot blind |  | Laboratory personnel blinded; low for laboratory confirmed outcomes and high for respiratory illnesses | Low | 376/380 clusters analyzed and reasons provided | Low | protocol published | Low | Not for-profit |

† Judgement applies to all outcomes unless specified otherwise
